# Hydrogen bonding, π–π stacking and van der Waals forces-dominated layered regions in the crystal structure of 4-amino­pyridinium hydrogen (9-phosphono­non­yl)phospho­nate

**DOI:** 10.1107/S2056989016014298

**Published:** 2016-09-23

**Authors:** Martin van Megen, Guido J. Reiss, Walter Frank

**Affiliations:** a Institut für Anorganische Chemie und Strukturchemie, Lehrstuhl II: Material- und Strukturforschung, Heinrich-Heine-Universität Düsseldorf, Universitätsstrasse 1, D-40225 Düsseldorf, Germany

**Keywords:** crystal structure, 4-amino­pyridinium, bis­(phospho­nate), hydrogen bonding

## Abstract

The asymmetric unit of the title mol­ecular salt, [C_5_H_7_N_2_^+^][(HO)_2_OP(CH_2_)_9_PO_2_(OH)^−^], consists of one 4-amino­pyridinium cation and one hydrogen (9-phos­phono­non­yl)phospho­nate anion, both in general positions. As expected, the 4-amino­pyridinium moieties are protonated exclusively at their endocyclic nitro­gen atom due to a mesomeric stabilization by the imine form which would not be given in the corresponding double-protonated dicationic species. In the crystal, the phosphonyl (–PO_3_H_2_) and hydrogen phospho­nate (–PO_3_H) groups of the anions form two-dimensional O—H⋯O hydrogen-bonded networks in the *ab* plane built from 24-membered hydrogen-bonded ring motifs with the graph-set descriptor *R* ^6^_6_(24). These networks are pairwise linked by the anions’ alkyl­ene chains. The 4-amino­pyridinium cations are stacked in parallel displaced face-to-face arrangements and connect neighboring anionic substructures *via* medium–strong charge-supported N—H⋯O hydrogen bonds along the *c* axis. The resulting three-dimensional hydrogen-bonded network shows clearly separated hydro­philic and hydro­phobic structural domains.

## Abstract 2

The structure of the title mol­ecular salt, [C_5_H_7_N_2_][(HO)_2_OP(CH_2_)_9_PO_2_(OH)], shows a three-dimensional network with hydrogen bonding, π–π stacking, and van der Waals forces-dominated layered regions.

## Chemical context   

Salts of organo­phospho­nic acids with organic cations, *e.g.* with protonated primary (Mahmoudkhani & Langer, 2002*b* ▸), secondary (Wheatley *et al.*, 2001 ▸) and tertiary amines (Kan & Ma, 2011 ▸) are of growing inter­est in supra­molecular chemistry and crystal engineering. Besides their inter­esting topologies and structural diversity, they seem to be feasible model compounds for metal phospho­nates as they exhibit similar structural characteristics but are less difficult to crystallize. Mostly, these organic solids establish extended hydrogen-bonded networks which are characterized by a rich diversity of strong charge-supported hydrogen bonds (Aakeröy & Seddon, 1993 ▸) and can either be one-, two- or three-dimensional. This contribution forms part of our research on the principles of the arrangement of alkane-*α*,*ω*-di­phospho­nic acids (van Megen *et al.*, 2015 ▸) and their organic aminium salts (van Megen *et al.*, 2016 ▸). Moreover, amino­pyridines and the related protonated cations are of crucial inter­est in the field of biochemistry (Muñoz-Caro & Niño, 2002 ▸; Bolliger *et al.*, 2011 ▸) and are also used as counter-cations to stabilize complex salts (Reiss & Leske, 2014*a* ▸,*b* ▸), in crystal engineering (Sertucha *et al.*, 1998 ▸; Surbella III *et al.*, 2016 ▸) as well as in polymer chemistry (Deng *et al.*, 2015 ▸).
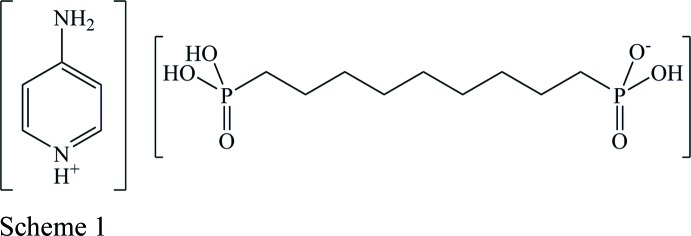


## Structural commentary   

**Figure 1:**
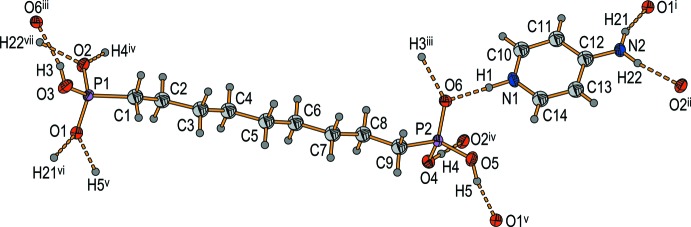
The asymmetric unit of the title compound plus symmetry-related hydrogen-bonded atoms [displacement ellipsoids are drawn at the 50% probability level; hydrogen atoms are drawn as spheres with arbitrary radii; symmetry codes: (i) 1 + *x*, −1 + *y*, 1 + *z*; (ii) *x*, −1 + *y*, 1 + *z*; (iii) 1 − *x*, 2 − *y*, 1 − *z*; (iv) 1 − *x*, 1 − *y*, 1 − *z*; (v) −*x*, 1 − *y*, 1 − *z*; (vi) −1 + *x*, 1 + *y*, −1 + *z*, (vii) *x*, 1 + *y*, −1 + *z*].

The asymmetric unit of the title compound, [C_5_H_7_N_2_^+^][(HO)_2_OP(CH_2_)_9_PO_2_(OH)^−^], consists of one 4-amino­pyridinium cation and one hydrogen (9-phosphono­non­yl)phospho­nate anion, both in general positions (Fig. 1 ▸). Generally, the first protonation of the 4-amino­pyridine can take place at the exo- as well as at the endocyclic nitro­gen atom. In the literature, all monoprotonated 4-amino­pyridines characterized to date are protonated at the endocyclic nitro­gen atom. Geometric parameters derived from the single-crystal diffraction experiment for the title compound show a short exocyclic N—C bond length [1.324 (2) Å] and slightly longer C—C and C—N bond lengths of the six-membered ring [1.350 (3)–1.425 (2) Å]. The bonding properties of this cation are best described by a pair of mesomeric structures: the enamine and the imine form (Scheme 2), which have been discussed in detail before (Koleva *et al.*, 2008 ▸).
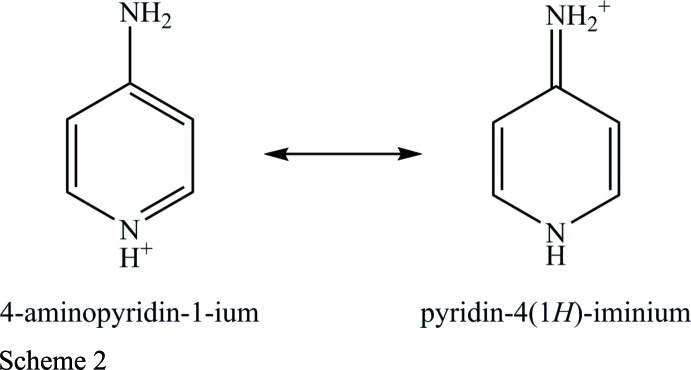


For the designation of the title compound, the systematic name of the amino form is used throughout this article. The bond lengths and angles of the anion are unexceptional and lie within the expected ranges. The alkyl­ene chain of the anion shows nearly anti­periplanar conformations. In detail, the P—OH distances of the phospho­nate moieties have values between 1.5535 (13) and 1.5786 (14) Å, longer than the P=O distances [1.5045 (13)–1.5149 (12) Å].

## Supra­molecular features   

**Figure 2:**
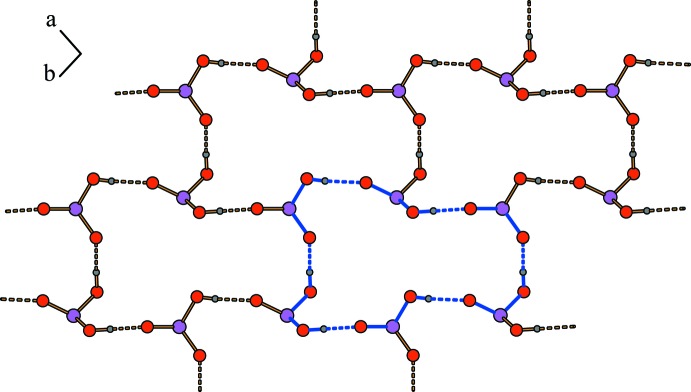
Two-dimensional hydrogen-bonded networks composed of phosphonyl and hydrogen phospho­nate groups. The graph set 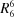
(24) is indicated by blue bonds.

**Figure 3:**
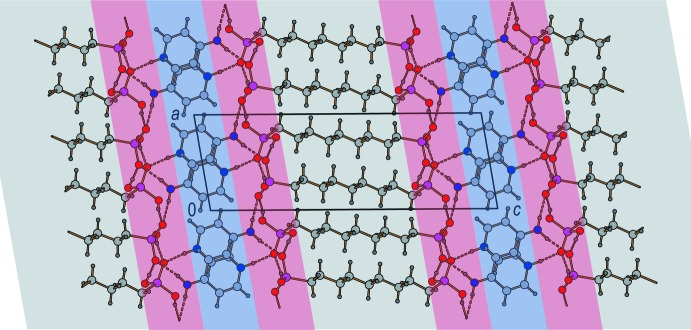
View along [010] of the title structure, showing the hydrogen bonding (red), π–π stacking (blue), and van der Waals forces (grey) dominated layered regions within the three-dimensional network.

Within the crystal of the title compound, the phosphonyl and hydrogen phospho­nate groups of the anions form two-dimensional O—H⋯O hydrogen-bonded networks which propagate in the *ab* plane. These networks contain 24-membered rings classified as a third level graph set 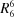
(24) (Etter *et al.*, 1990 ▸; Fig. 2 ▸; Table 1 ▸). 24-Membered hydrogen-bonded rings have been well known for decades (e.g. Mootz & Poll, 1984 ▸). In particular, the 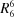
(24) motif is very common (*e.g*. Gomathi & Mu­thiah, 2011 ▸; Maspoch *et al.*, 2007 ▸). Along the *c*-axis direction, these networks are pairwise linked by the anions’ alkyl­ene chains to form a three-dimensional anionic substructure. The 4-amino­pyridinium cations show π–π stacking inter­actions. The rings are oriented in parallel displaced face-to-face arrangements (Grimme, 2008 ▸; Fig. 3 ▸). The geometry of these π–π inter­actions is reflected by distances of 3.25 and 3.32 Å between neighbouring pyridinium rings and centroid offsets of 2.37 and 2.42 Å. These findings are comparable to those found for other compounds containing pyridyl moieties (Janiak, 2000 ▸). Anions and cations are connected by medium–strong, charge-supported N—H⋯O hydrogen bonds (Steiner, 2002 ▸; Table 2 ▸) along the *c* axis. For these connections, each nitro­gen-bound hydrogen atom forms one unbifurcated hydrogen bond (Fig. 1 ▸). The resulting three-dimensional hydrogen-bonded network clearly shows separated hydro­philic and hydro­phobic regions (Fig. 3 ▸).

## Related structures   

For related phospho­nate and bis­(phospho­nate) salts, see: Ferguson *et al.* (1998 ▸); Fu *et al.* (2004 ▸); Fuller & Heimer (1995 ▸); Glidewell *et al.* (2000 ▸); Kan & Ma (2011 ▸); Mahmoudkhani & Langer (2002*a* ▸,*b* ▸,*c* ▸); van Megen *et al.* (2016 ▸); Plabst *et al.* (2009 ▸); Wheatley *et al.* (2001 ▸). For related 4-amino­pyridinium salts, see: Sertucha *et al.* (1998 ▸); Reiss & Leske (2014*a* ▸,*b* ▸); Surbella III *et al.* (2016 ▸).

## Synthesis and crystallization   

**Figure 4:**
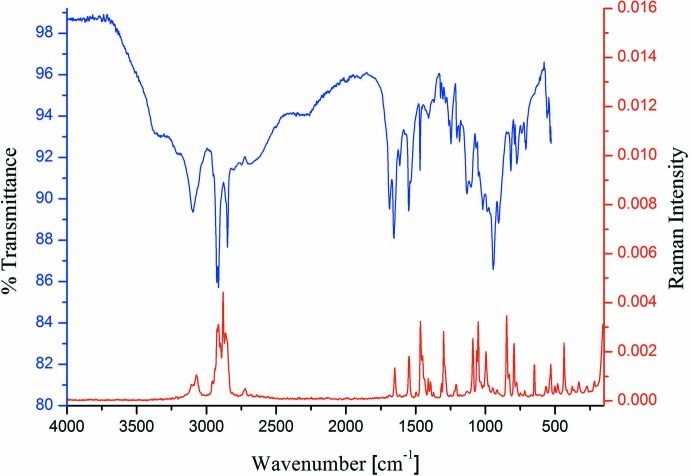
The IR (blue) and Raman (red) spectra of the title compound.

Equimolar qu­anti­ties (0.5 mmol) of 4-amino­pyridine (47.1 mg) and nonane-1,9-di­phospho­nic acid (144.1 mg) were dissolved in methanol, separately. The solutions were mixed and stored in an open petri dish. Within several days, colorless platelet-shaped crystals of the title compound were obtained by slow evaporation of the solvent. 4-Amino­pyridine was purchased from commercial sources and nonane-1,9-di­phospho­nic acid was synthesized according to the literature (Schwarzenbach & Zurc, 1950 ▸; Moedritzer & Irani, 1961 ▸; Griffith *et al.*, 1998 ▸). Elemental analysis: C_14_H_28_N_2_O_6_P_2_ (382.3): calculated C 44.0, H 7.4, N 7.3; found C 43.6, H 7.9, N 7.1. M. p.: 157 °C. The IR and Raman spectra of the title compound are shown in Fig. 4 ▸.

## Refinement   

Crystal data, data collection and structure refinement details are summarized in Table 2 ▸. All hydrogen atoms bound to either nitro­gen or oxygen atoms were identified in difference syntheses and refined without any geometric constraints or restraints with individual *U*_iso_(H) values. Carbon-bound hydrogen atoms were included using a riding model (AFIX23 option of the *SHELX* program for the methyl­ene groups and AFIX43 option for the methine groups).

## Supplementary Material

Crystal structure: contains datablock(s) I, publication_text. DOI: 10.1107/S2056989016014298/hb7610sup1.cif

Structure factors: contains datablock(s) I. DOI: 10.1107/S2056989016014298/hb7610Isup2.hkl

Supporting information file. DOI: 10.1107/S2056989016014298/hb7610Isup3.cml

CCDC reference: 1503436

Additional supporting information: crystallographic information; 3D view; checkCIF report

## Figures and Tables

**Table 1:** Hydrogen-bond geometry (Å, °)

*D*—H⋯*A*	*D*—H	H⋯*A*	*D*⋯*A*	*D*—H⋯*A*
O3—H3⋯O6^i^	0.78 (3)	1.85 (3)	2.6171 (18)	166 (3)
O5—H5⋯O1^ii^	0.88 (3)	1.64 (3)	2.5059 (18)	168 (3)
O4—H4⋯O2^iii^	0.91 (3)	1.59 (3)	2.4977 (17)	178 (3)
N1—H1⋯O6	0.96 (2)	1.74 (3)	2.696 (2)	173 (2)
N2—H22⋯O2^iv^	0.90 (3)	1.92 (3)	2.806 (2)	170 (2)
N2—H21⋯O1^v^	0.88 (3)	2.14 (3)	2.965 (2)	156 (3)

Symmetry codes: (i) 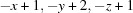; (ii) 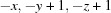; (iii) 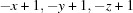; (iv) 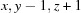; (v) 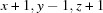.

**Table 2:** Experimental details

Crystal data	
Chemical formula	C_5_H_7_N_2_^+^·C_9_H_21_O_6_P_2_^−^
*M*_r_	382.32
Crystal system, space group	Triclinic, *P* 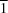
Temperature (K)	123
*a*, *b*, *c* (Å)	6.7275 (4), 6.8963 (4), 20.0643 (10)
α, β, γ (°)	97.956 (4), 98.767 (4), 94.309 (5)
*V* (Å^3^)	906.73 (9)
*Z*	2
Radiation type	Mo *K*α
μ (mm^−1^)	0.27
Crystal size (mm)	0.33 × 0.07 × 0.03

Data collection	
Diffractometer	Stoe IPDS
No. of measured, independent and observed [*I* > 2σ(*I*)] reflections	8855, 4131, 3674
*R*_int_	0.029
(sin θ/λ)_max_ (Å^−1^)	0.650

Refinement	
*R*[*F* ^2^ > 2σ(*F* ^2^)], *wR*(*F* ^2^), *S*	0.038, 0.079, 1.02
No. of reflections	4131
No. of parameters	241
H-atom treatment	H atoms treated by a mixture of independent and constrained refinement
Δρ_max_, Δρ_min_ (e Å^−3^)	0.50, −0.36

Computer programs: *X-AREA* (Stoe & Cie, 2002 ▸), *SHELXT* (Sheldrick, 2015*a* ▸), *SHELXL-2014/7* (Sheldrick, 2015*b* ▸) and *DIAMOND* (Brandenburg, 2015 ▸).

## supplementary crystallographic information

### Crystal data

**Table:**

C_5_H_7_N_2_^+^·C_9_H_21_O_6_P_2_^−^	*Z* = 2
*M**_r_* = 382.32	*F*(000) = 408
Triclinic, *P*1	*D*_x_ = 1.400 Mg m^−^^3^
*a* = 6.7275 (4) Å	Mo *K*α radiation, λ = 0.71073 Å
*b* = 6.8963 (4) Å	Cell parameters from 6853 reflections
*c* = 20.0643 (10) Å	θ = 3.0–35.3°
α = 97.956 (4)°	µ = 0.27 mm^−^^1^
β = 98.767 (4)°	*T* = 123 K
γ = 94.309 (5)°	Platelet, colourless
*V* = 906.73 (9) Å^3^	0.33 × 0.07 × 0.03 mm

### Data collection

**Table:**

Stoe IPDS diffractometer	*R*_int_ = 0.029
Radiation source: sealed tube	θ_max_ = 27.5°, θ_min_ = 3.0°
ω scans	*h* = −8→8
8855 measured reflections	*k* = −8→8
4131 independent reflections	*l* = −26→26
3674 reflections with *I* > 2σ(*I*)	

### Refinement

**Table:**

Refinement on *F*^2^	Secondary atom site location: difference Fourier map
Least-squares matrix: full	Hydrogen site location: mixed
*R*[*F*^2^ > 2σ(*F*^2^)] = 0.038	H atoms treated by a mixture of independent and constrained refinement
*wR*(*F*^2^) = 0.079	*w* = 1/[σ^2^(*F*_o_^2^) + (0.011*P*)^2^ + 1.110*P*] where *P* = (*F*_o_^2^ + 2*F*_c_^2^)/3
*S* = 1.02	(Δ/σ)_max_ = 0.001
4131 reflections	Δρ_max_ = 0.50 e Å^−^^3^
241 parameters	Δρ_min_ = −0.36 e Å^−^^3^
0 restraints	

### Special details

**Table:**

Geometry. All esds (except the esd in the dihedral angle between two l.s. planes) are estimated using the full covariance matrix. The cell esds are taken into account individually in the estimation of esds in distances, angles and torsion angles; correlations between esds in cell parameters are only used when they are defined by crystal symmetry. An approximate (isotropic) treatment of cell esds is used for estimating esds involving l.s. planes.

### Fractional atomic coordinates and isotropic or equivalent isotropic displacement parameters (Å^2^)

**Table:**

	*x*	*y*	*z*	*U*_iso_*/*U*_eq_	
P1	0.29655 (6)	1.15924 (6)	0.22320 (2)	0.01579 (10)	
O1	0.14667 (19)	1.0094 (2)	0.17549 (6)	0.0230 (3)	
N1	0.5995 (3)	0.2836 (2)	0.92948 (8)	0.0257 (3)	
H1	0.553 (4)	0.306 (4)	0.8838 (13)	0.036 (6)*	
C1	0.2470 (3)	1.1616 (3)	0.30883 (8)	0.0179 (3)	
H1A	0.3161	1.2802	0.3371	0.021*	
H1B	0.1032	1.1663	0.3086	0.021*	
P2	0.24757 (6)	0.29013 (6)	0.77082 (2)	0.01589 (10)	
O2	0.51802 (18)	1.13618 (18)	0.22159 (6)	0.0199 (3)	
N2	0.7740 (3)	0.1858 (3)	1.12583 (8)	0.0252 (3)	
H21	0.901 (4)	0.169 (4)	1.1403 (14)	0.049 (8)*	
H22	0.681 (4)	0.172 (4)	1.1527 (12)	0.035 (6)*	
C2	0.3143 (3)	0.9827 (3)	0.34082 (8)	0.0191 (3)	
H2A	0.2686	0.8640	0.3084	0.023*	
H2B	0.4608	0.9929	0.3504	0.023*	
O3	0.2486 (2)	1.3660 (2)	0.20293 (7)	0.0239 (3)	
C3	0.2306 (3)	0.9668 (3)	0.40681 (8)	0.0191 (3)	
H3A	0.2699	1.0891	0.4379	0.023*	
H3B	0.0842	0.9503	0.3964	0.023*	
H3	0.343 (5)	1.442 (5)	0.2068 (17)	0.070 (11)*	
O4	0.24107 (19)	0.08343 (18)	0.72796 (6)	0.0188 (2)	
C4	0.3028 (3)	0.7975 (3)	0.44244 (8)	0.0195 (3)	
H4A	0.4490	0.8157	0.4542	0.023*	
H4B	0.2664	0.6752	0.4112	0.023*	
H4	0.330 (5)	0.006 (5)	0.7471 (16)	0.066 (9)*	
O5	0.1083 (2)	0.27587 (19)	0.82576 (6)	0.0216 (3)	
C5	0.2125 (3)	0.7822 (3)	0.50710 (9)	0.0195 (3)	
H5A	0.2483	0.9055	0.5379	0.023*	
H5B	0.0664	0.7648	0.4950	0.023*	
H5	0.017 (5)	0.174 (5)	0.8191 (16)	0.064 (9)*	
O6	0.45679 (19)	0.37295 (19)	0.80557 (6)	0.0220 (3)	
C6	0.2806 (3)	0.6151 (3)	0.54491 (8)	0.0188 (3)	
H6A	0.4264	0.6325	0.5579	0.023*	
H6B	0.2450	0.4910	0.5146	0.023*	
C7	0.1838 (3)	0.6074 (3)	0.60867 (8)	0.0179 (3)	
H7A	0.0383	0.5858	0.5951	0.022*	
H7B	0.2146	0.7341	0.6377	0.022*	
C8	0.2526 (3)	0.4476 (3)	0.65035 (8)	0.0180 (3)	
H8A	0.3972	0.4716	0.6661	0.022*	
H8B	0.2261	0.3205	0.6214	0.022*	
C9	0.1432 (3)	0.4431 (3)	0.71211 (8)	0.0175 (3)	
H9A	0.0022	0.3966	0.6959	0.021*	
H9B	0.1488	0.5762	0.7360	0.021*	
C10	0.7973 (3)	0.2769 (3)	0.95325 (10)	0.0283 (4)	
H10	0.8919	0.2928	0.9247	0.034*	
C11	0.8604 (3)	0.2470 (3)	1.01867 (9)	0.0272 (4)	
H11	0.9972	0.2420	1.0341	0.033*	
C12	0.7189 (3)	0.2236 (3)	1.06310 (9)	0.0199 (3)	
C13	0.5127 (3)	0.2384 (3)	1.03659 (9)	0.0214 (4)	
H13	0.4144	0.2288	1.0642	0.026*	
C14	0.4601 (3)	0.2665 (3)	0.97075 (10)	0.0242 (4)	
H14	0.3249	0.2741	0.9537	0.029*	

### Atomic displacement parameters (Å^2^)

**Table:**

	*U*^11^	*U*^22^	*U*^33^	*U*^12^	*U*^13^	*U*^23^
P1	0.01293 (19)	0.0198 (2)	0.0158 (2)	0.00022 (16)	0.00233 (15)	0.00733 (16)
O1	0.0224 (6)	0.0286 (7)	0.0167 (6)	−0.0056 (5)	0.0023 (5)	0.0042 (5)
N1	0.0360 (9)	0.0236 (8)	0.0159 (7)	0.0009 (7)	−0.0008 (6)	0.0036 (6)
C1	0.0179 (8)	0.0202 (8)	0.0161 (8)	−0.0003 (6)	0.0038 (6)	0.0048 (6)
P2	0.0175 (2)	0.0167 (2)	0.01360 (19)	−0.00040 (16)	0.00111 (15)	0.00535 (15)
O2	0.0163 (6)	0.0239 (6)	0.0228 (6)	0.0042 (5)	0.0057 (5)	0.0111 (5)
N2	0.0187 (8)	0.0392 (10)	0.0194 (7)	0.0036 (7)	0.0035 (6)	0.0097 (7)
C2	0.0185 (8)	0.0237 (9)	0.0162 (8)	0.0015 (7)	0.0030 (6)	0.0072 (6)
O3	0.0158 (6)	0.0267 (7)	0.0328 (7)	0.0026 (5)	0.0047 (5)	0.0162 (6)
C3	0.0195 (8)	0.0229 (9)	0.0163 (8)	0.0003 (7)	0.0047 (6)	0.0066 (6)
O4	0.0190 (6)	0.0194 (6)	0.0178 (6)	0.0029 (5)	0.0014 (5)	0.0036 (5)
C4	0.0201 (8)	0.0239 (9)	0.0161 (8)	0.0009 (7)	0.0038 (6)	0.0079 (6)
O5	0.0280 (7)	0.0206 (6)	0.0170 (6)	−0.0021 (5)	0.0070 (5)	0.0041 (5)
C5	0.0209 (8)	0.0217 (8)	0.0169 (8)	0.0002 (7)	0.0039 (6)	0.0063 (6)
O6	0.0216 (6)	0.0228 (6)	0.0202 (6)	−0.0045 (5)	−0.0032 (5)	0.0091 (5)
C6	0.0193 (8)	0.0221 (8)	0.0161 (8)	0.0008 (7)	0.0041 (6)	0.0056 (6)
C7	0.0195 (8)	0.0199 (8)	0.0153 (7)	0.0014 (6)	0.0029 (6)	0.0060 (6)
C8	0.0191 (8)	0.0200 (8)	0.0157 (7)	0.0019 (6)	0.0027 (6)	0.0058 (6)
C9	0.0178 (8)	0.0198 (8)	0.0157 (7)	0.0020 (6)	0.0027 (6)	0.0054 (6)
C10	0.0305 (10)	0.0338 (11)	0.0206 (9)	0.0008 (8)	0.0077 (7)	0.0020 (8)
C11	0.0206 (9)	0.0390 (11)	0.0215 (9)	0.0018 (8)	0.0038 (7)	0.0032 (8)
C12	0.0212 (8)	0.0192 (8)	0.0184 (8)	0.0008 (7)	0.0025 (6)	0.0014 (6)
C13	0.0206 (8)	0.0207 (8)	0.0232 (9)	0.0010 (7)	0.0039 (7)	0.0047 (7)
C14	0.0249 (9)	0.0206 (9)	0.0254 (9)	0.0022 (7)	−0.0020 (7)	0.0046 (7)

### Geometric parameters (Å, º)

**Table:**

P1—O1	1.5088 (13)	C4—H4A	0.9700
P1—O2	1.5149 (12)	C4—H4B	0.9700
P1—O3	1.5786 (14)	O5—H5	0.88 (3)
P1—C1	1.7974 (17)	C5—C6	1.526 (2)
N1—C10	1.350 (3)	C5—H5A	0.9700
N1—C14	1.352 (3)	C5—H5B	0.9700
N1—H1	0.96 (2)	C6—C7	1.527 (2)
C1—C2	1.534 (2)	C6—H6A	0.9700
C1—H1A	0.9700	C6—H6B	0.9700
C1—H1B	0.9700	C7—C8	1.530 (2)
P2—O6	1.5045 (13)	C7—H7A	0.9700
P2—O4	1.5535 (13)	C7—H7B	0.9700
P2—O5	1.5601 (13)	C8—C9	1.537 (2)
P2—C9	1.7880 (17)	C8—H8A	0.9700
N2—C12	1.324 (2)	C8—H8B	0.9700
N2—H21	0.88 (3)	C9—H9A	0.9700
N2—H22	0.90 (3)	C9—H9B	0.9700
C2—C3	1.530 (2)	C10—C11	1.365 (3)
C2—H2A	0.9700	C10—H10	0.9300
C2—H2B	0.9700	C11—C12	1.415 (3)
O3—H3	0.78 (3)	C11—H11	0.9300
C3—C4	1.523 (2)	C12—C13	1.425 (2)
C3—H3A	0.9700	C13—C14	1.359 (3)
C3—H3B	0.9700	C13—H13	0.9300
O4—H4	0.91 (3)	C14—H14	0.9300
C4—C5	1.527 (2)		
			
O1—P1—O2	116.41 (8)	C6—C5—C4	114.77 (15)
O1—P1—O3	105.96 (8)	C6—C5—H5A	108.6
O2—P1—O3	108.76 (7)	C4—C5—H5A	108.6
O1—P1—C1	109.09 (8)	C6—C5—H5B	108.6
O2—P1—C1	109.62 (7)	C4—C5—H5B	108.6
O3—P1—C1	106.51 (8)	H5A—C5—H5B	107.6
C10—N1—C14	120.49 (16)	C5—C6—C7	112.06 (14)
C10—N1—H1	121.8 (15)	C5—C6—H6A	109.2
C14—N1—H1	117.6 (15)	C7—C6—H6A	109.2
C2—C1—P1	113.66 (12)	C5—C6—H6B	109.2
C2—C1—H1A	108.8	C7—C6—H6B	109.2
P1—C1—H1A	108.8	H6A—C6—H6B	107.9
C2—C1—H1B	108.8	C6—C7—C8	114.51 (14)
P1—C1—H1B	108.8	C6—C7—H7A	108.6
H1A—C1—H1B	107.7	C8—C7—H7A	108.6
O6—P2—O4	113.40 (7)	C6—C7—H7B	108.6
O6—P2—O5	109.15 (7)	C8—C7—H7B	108.6
O4—P2—O5	108.70 (7)	H7A—C7—H7B	107.6
O6—P2—C9	111.12 (8)	C7—C8—C9	111.67 (14)
O4—P2—C9	105.43 (8)	C7—C8—H8A	109.3
O5—P2—C9	108.89 (8)	C9—C8—H8A	109.3
C12—N2—H21	119.9 (18)	C7—C8—H8B	109.3
C12—N2—H22	119.6 (16)	C9—C8—H8B	109.3
H21—N2—H22	120 (2)	H8A—C8—H8B	107.9
C3—C2—C1	112.06 (14)	C8—C9—P2	113.70 (12)
C3—C2—H2A	109.2	C8—C9—H9A	108.8
C1—C2—H2A	109.2	P2—C9—H9A	108.8
C3—C2—H2B	109.2	C8—C9—H9B	108.8
C1—C2—H2B	109.2	P2—C9—H9B	108.8
H2A—C2—H2B	107.9	H9A—C9—H9B	107.7
P1—O3—H3	115 (2)	N1—C10—C11	120.95 (18)
C4—C3—C2	113.89 (15)	N1—C10—H10	119.5
C4—C3—H3A	108.8	C11—C10—H10	119.5
C2—C3—H3A	108.8	C10—C11—C12	120.38 (18)
C4—C3—H3B	108.8	C10—C11—H11	119.8
C2—C3—H3B	108.8	C12—C11—H11	119.8
H3A—C3—H3B	107.7	N2—C12—C11	121.92 (17)
P2—O4—H4	113 (2)	N2—C12—C13	121.32 (17)
C3—C4—C5	112.68 (15)	C11—C12—C13	116.75 (16)
C3—C4—H4A	109.1	C14—C13—C12	119.77 (17)
C5—C4—H4A	109.1	C14—C13—H13	120.1
C3—C4—H4B	109.1	C12—C13—H13	120.1
C5—C4—H4B	109.1	N1—C14—C13	121.60 (18)
H4A—C4—H4B	107.8	N1—C14—H14	119.2
P2—O5—H5	117 (2)	C13—C14—H14	119.2
			
O1—P1—C1—C2	74.30 (14)	O6—P2—C9—C8	66.99 (14)
O2—P1—C1—C2	−54.24 (14)	O4—P2—C9—C8	−56.25 (14)
O3—P1—C1—C2	−171.74 (12)	O5—P2—C9—C8	−172.75 (12)
P1—C1—C2—C3	−167.86 (12)	C14—N1—C10—C11	1.9 (3)
C1—C2—C3—C4	−176.88 (14)	N1—C10—C11—C12	−0.4 (3)
C2—C3—C4—C5	−178.52 (15)	C10—C11—C12—N2	176.98 (19)
C3—C4—C5—C6	−179.83 (15)	C10—C11—C12—C13	−1.7 (3)
C4—C5—C6—C7	−179.62 (14)	N2—C12—C13—C14	−176.38 (18)
C5—C6—C7—C8	−177.72 (15)	C11—C12—C13—C14	2.3 (3)
C6—C7—C8—C9	−177.78 (14)	C10—N1—C14—C13	−1.3 (3)
C7—C8—C9—P2	−169.70 (12)	C12—C13—C14—N1	−0.9 (3)

### Hydrogen-bond geometry (Å, º)

**Table:**

*D*—H···*A*	*D*—H	H···*A*	*D*···*A*	*D*—H···*A*
O3—H3···O6^i^	0.78 (3)	1.85 (3)	2.6171 (18)	166 (3)
O5—H5···O1^ii^	0.88 (3)	1.64 (3)	2.5059 (18)	168 (3)
O4—H4···O2^iii^	0.91 (3)	1.59 (3)	2.4977 (17)	178 (3)
N1—H1···O6	0.96 (2)	1.74 (3)	2.696 (2)	173 (2)
N2—H22···O2^iv^	0.90 (3)	1.92 (3)	2.806 (2)	170 (2)
N2—H21···O1^v^	0.88 (3)	2.14 (3)	2.965 (2)	156 (3)

Symmetry codes: (i) −*x*+1, −*y*+2, −*z*+1; (ii) −*x*, −*y*+1, −*z*+1; (iii) −*x*+1, −*y*+1, −*z*+1; (iv) *x*, *y*−1, *z*+1; (v) *x*+1, *y*−1, *z*+1.
